# The genetic background of antibiotic resistance among clinical uropathogenic *Escherichia coli* strains

**DOI:** 10.1007/s11033-018-4254-0

**Published:** 2018-07-14

**Authors:** Wioletta Adamus-Białek, Anna Baraniak, Monika Wawszczak, Stanisław Głuszek, Beata Gad, Klaudia Wróbel, Paulina Bator, Marta Majchrzak, Paweł Parniewski

**Affiliations:** 1 Department of Surgery and Surgical Nursery with a Genetics Laboratory, Faculty of Medicine and Health Sciences, Jan Kochanowski University, IX Wieków Kielc 19A Av., 25-317 Kielce, Poland; 2 National Medicines Institute, Chełmska 30/34, 00-725 Warsaw, Poland; 3 Institute of Medical Biology PAS, Lodowa 106, 93-232 Lodz, Poland

**Keywords:** UPEC, Antibiotic resistance, Beta-lactamases, Quinolones

## Abstract

The spreading mechanisms of antibiotic resistance are related to many bacterial and environment factors. The overuse of antibiotics is leading to an unceasing emergence of new multidrug resistant strains. This problem also concerns uropathogenic *Escherichia coli* strains, which is the most common pathogen causing urinary tract infections. The aim of this study was the genetic analysis of antibiotic resistance in comparison to the phenotypic background of *E. coli* strains. The characterized collection of *E. coli* strains isolated 10 years ago from the urine samples of patients with urinary tract infections was used for antimicrobial susceptibility testing (the disc diffusion method) and analysis of antibiotic resistance genes (PCR reaction, sequencing). Additionally, the presence of ESBL strains was analyzed. Fourteen genes were associated with resistance to beta-lactams, aminoglycosides, sulfonamides and quinolones. The genetic analysis revealed that *bla*_TEM-1_ and *sul2* were present in almost all of the studied strains. Other drug-resistance genes were very rare or non-existent. Otherwise, the phenotypic resistance to fluoroquinolones was well correlated with the genotypic background of the studied bacteria. The presence of particular genes and specific mutations indicate a high bacterial potential to multidrug resistance. On the other hand, it needs to be emphasized that the standard disk diffusion test for the routine antimicrobial susceptibility analysis is still the best way to estimate the current situation of bacterial drug-resistance.

## Introduction

Molecular analyses are increasingly being introduced into routine diagnostics. The identification of the genetic determinants of pathogenicity and antibiotic resistance is very important for prevention against the widespread of hazardous bacteria, especially Multiple Drug Resistant (MDR) strains [1]. In the case of urinary tract infections, where uropathogenic *Escherichia coli* strains (UPEC) are the main causative agent [2], the most attention is paid to beta-lactam antibiotics [3]. Among resistance genes that are often located on plasmids are those coding for multiple types of β-lactamases (*bla* genes) [4, 5]. In a significant part, it refers to extended-spectrum β-lactamases (ESBLs) that are one of the main problems in the epidemiology of infections caused by organisms from the family *Enterobacteriaceae*. ESBLs usually confer resistance to all penicillins, cephalosporins (except for cephamycins), and monobactams, being inhibited by β-lactam inhibitors [6, 7], and they are the predominant source of enterobacterial resistance to 3rd- and 4th-generation cephalosporins [8, 9]. Among ESBLs observed in *E. coli*, the most commonly identified are enzymes from the family CTX-M (mostly CTX-M-1 lineage), followed by SHVs, and recently the less and less frequent TEMs [10–13]. Another group of acquired β-lactamases responsible for resistance to newer generation β-lactams are cephalosporinases of the AmpC type [14]. These are derivatives of enzymes specific for organisms like *Enterobacter cloacae, Citrobacter freundii* or *Morganella morganii* [15]. In general they confer a similar resistance profile to ESBLs, except for resistance to cephamycins but not 4th-generation cephalosporins, and resistance to β-lactam-inhibitor combinations, mainly those with clavulanic acid [8]. Among the several families of the acquired AmpC-type enzymes identified so far, the group deriving from *C. freundii* is the largest, including CMY-2 which is the most common enzyme of this kind [16, 17].

Similarly, the *E. coli* resistance to other antibiotics like sulfonamides and aminoglycosides applied during UTI treatment is often associated with the presence of specific plasmids. Resistance to sulfonamides is determined by three genes (*sul1, sul2* and *sul3*) [18]. Gene s*ul1* has usually been identified on large conjugative plasmids, opposite *sul2* has mainly been detected on small non-conjugative plasmids. Recently, sul2 has been observed also on a large conjugative plasmids related to the streptomycin resistance [18–20]. *sul3* is the least known and also the least frequently detected plasmid gene in *E. coli* [19]. At present, the knowledge about various *sul* genes and their carriers is poor and diverse depending on reservoirs (e.g. animals and human) [21]. Similarly, aminoglycosides resistance is also connected with few genes carried by plasmids. The *aadB, aac(3)-II* and *aac(3)-IV* genes are related to the gentamycin, tobramycin, neomycin resistance and other aminoglycosides. They belong to the most frequent genes detected in *Escherichia coli* strains and other Gram-negative bacteria [22].

The frequent antibiotic resistance of UPEC strains is also associated with fluoroquinolones. Primarily, they induce the mutation in DNA gyrase (*gyrA*) and topoisomerase IV (*parC*) genes. In the literature, the mutations of genes controlling fluoroquinolones accumulation are also describing [23]. Additionally, resistance to fluoroquinolones can also be facilitated by plasmids producing the Qnr protein (QnrA, QnrB, QnrS), which protects the antibiotic targets from quinolone treatment. Qnr plasmids induce resistance on low level, but it was observed also that their presence strongly enhance the quinolone resistance determined by other mechanisms [24, 25].

The uninterrupted increasing of resistance and the emergence of MDR strains are still monitored among UPEC [26]. Therefore, there is a need for periodic screening of common bacterial pathogens such as UPEC to control their antibiotic susceptibility profiles in different communities [27, 28]. It seems to be important to also monitor the distribution of genes associated with antibiotic resistance. This knowledge can allow us to prevent the spreading of strains with a high risk of MDR expression. In reference to this, the aim of the study was to investigate the prevalence of the genes encoding the resistance to the most popular antibiotics class (beta-lactams, aminoglycosides, sulfonamides and quinolones) used during therapy of UTI against *E. coli* [24, 26, 29].

## Materials and methods

### Bacterial strains

A previously characterized collection of 127 clinical *Escherichia coli* strains [30, 31] isolated from the urine of patients in different hospital wards in Lodz (Poland) in the years 2005–2007 was used. Additionally, reference *E. coli* strains producing different beta-lactamases (No. 3272/96–TEM-1, No. 3290/96–CTX-M-3, No. 3274/96–SHV-5, No. 394/06–CMY-2, No. 348/04–OXA-1) from the National Medicines Institute (Poland) and *E. coli* ATCC 25922 (Argenta) were used as a control during the antimicrobial disc diffusion test.

### Susceptibility testing and phenotypic ESBL detection

Antimicrobial susceptibility testing was performed using the disk-diffusion method on Mueller–Hinton agar, using commercial disks (Oxoid, Wesel, Germany). The isolates were tested against 16 antimicrobials: amoxicillin (AMX, 25 µg), amoxicillin/clavulanate (AMC, 30 µg), piperacillin (PIP, 30 µg), cefoxitin (FOX, 30 µg), cefotaxime (CTX, 5 µg), ceftazidime (CAZ, 10 µg), imipenem (IMP, 10 µg), amikacin (AMK, 30 µg), tobramycin (TOB, 10 µg), gentamicin (GEN, 10 µg), netilmicin (NET, 10 µg), norfloxacin (NOR, 10 µg), ciprofloxacin (CIP, 5 µg), ofloxacin (OFX, 5 µg), trimethoprim (TMP, 5 µg), trimethoprim-sulfamethoxazole (STX, 25 µg). The results of susceptibility testing were interpreted according to the European Committee on Antimicrobial Susceptibility Testing (EUCAST) guidelines [32]. *E. coli* ATCC 25922 was used as a quality control strain. Resistance to newer generation cephalosporins was also confirmed on chromID® ESBL plates (bioMérieux). Additionally, all isolates were tested for ESBL production by the double-disk synergy test (DDST) with disks containing cefotaxime, ceftazidime, and amoxicillin with clavulanate [33, 34].

### PCR detection of resistance genes

Bacterial DNA was purified with the Gen Elute™ Bacterial Genomic DNA kit (Sigma Aldrich, Germany). The identification of the genes was carried out by PCR using previously described primers. The specific PCR parameters for all primers used in the study and their references have been shown in Table 1. In this study *bla*_CTX-M-1_-, *bla*_TEM_-, *bla*_SHV_-, *bla*_OXA-1_-, and *bla*_CMY-2_-like β-lactamase-encoding genes and also *aac(3)-II, sul1, sul2*, and *sul3* were detected. The identification of the quinolones resistance was performed by PCR reaction for the *qnrA, qnrB, qnrS* genes detection and DNA sequencing of the PCR products of the *gyrA* and *parC g*enes. The individual adjusted conditions of DNA amplification were carried out for each gene. After PCR amplification, products were visualized under the gel documentation system. The nucleic acid sequences of PCR products (Macrogen Europe) were compared to the original gene sequences accessed in the GenBank of the National Center for Biotechnology Information (NCBI) database. Nucleotide and amino-acid sequences were analyzed by searching the GenBank database of the NCBI with the Basic Local Alignment Search Tool (BLAST network service).

**Table 1:** Oligonucleotides used in the study

Primer	Sequence (5′ → 3′)	Locus	Ta [°C]	PCR [bp]	Ref.
TEM-A TEM-B	ATAAAATTCTTGAAGAC TTACCAATGCTTAATCA	Flank of *bla*_TEM_-like genes	42	1181	[12]
P1C P2D	TTAATTCGTCTCTTCCAGA CAGCGCTTTTGCCGTCTAAG	Flank of *bla*_CTX-M1_-like genes	55	1042	[11]
SHV-A SHV-B	ACTGAATGAGGCGCTTCC ATCCCGCAGATAAATCACC	Flank of *bla*_SHV_-like genes	55	329	[12]
OXA-1/F OXA-1/R	ATGAAAAACACAATACATATCAAC TTTCCTGTAAGTGCGGACAC	Internal fragment of *bla*_OXA-1_-related genes	48	755	[35]
CF-1 CF-2	ATGATGAAAAAATCGATATG TTATTGCAGTTTTTCAAGAATG	Flank of *bla*_CMY_-like genes	45	1146	[15]
aac(3)-IIF aac(3)-II	TGAAACGCTGACGGAGCCTC GTCGAACAGGTAGCACTGAG	*aac(3)-II*	55	369	[36]
sul1-F sul1-R	TGGTGACGGTGTTCGGCATTC GCGAAGGTTTCCGAGAAGGTG	*sulI*	56	790	[37]
SUL2F SUL2R	CGGCATCGTCAACATAACCT TGTGCGGATGAAGTCAGCTC	*sulII*	55	721	[38]
SUL3F SUL3R	CAACGGAAGTGGGCGTTGTGGA GCTGCACCAATTCGCTGAACG	*sulIII*	57	244	[38]
gyrA-P1 gyrA-P3	TGT CCG AGA TGG CCT GAA GC TGC CGT CAT AGT TAT CAA CGA	QRDR *gyrA*	58	374	[37]
parC-3 parC-4	CCG TGC GTT GCC GTT TAT TG AAGTGCCGTCGAAGTTTGGCA	QRDR *parC*	58	368	[37]
qnrA-1 qnrA-2	ATTTCTCACGCCAGGATTTG GATCGGCAAAGGTTAGGTCA	*qnrA*	Gradient	516	[39, 40]
qnrB-1 qnrB-2	GATCGTGAAAGCCAGAAAGG ACGATGCCTGGTAGTTGTCC	*qnrB*		469	
qnrS-1 qnrS-2	ACGACATTCGTCAACTGCAA TAAATTGGCACCCTGTAGGC	*qnrS*		417	

*Ta* annealing temperature of PCR, *Ref* references, *bp* base pair

## Results

The collection of uropathogenic *Escherichia coli* strains was re-characterized based on antimicrobial susceptibility testing in order to verify the resistance profiles published previously [31]. Additionally, all isolates were tested for ESBL production. The whole collection of *E. coli* strains was analyzed based on the genetic conditioning of antibiotic resistance. The 5 genes encoding beta-lactamases (*bla*_TEM_, *bla*_CTX-M-1_, *bla*_SHV_, *bla*_OXA-1_, *bla*_CMY_) were detected at first in the reference group of *E. coli* ESBL strains and next, their presence was defined in the entire collection of clinical *E. coli* strains. Additionally, the presence of *aac(3)-II* determines resistance to aminoglycosides and three genes (*sul1, sul2, sul3*) encoding resistance to sulfonamides were studied.. Further, the strains resistant to quinolones [55] and the control representative group of strains [33]—intermediate sensitive and sensitive to quinolones—were analyzed based on the mutations in *parC* and *gyrA* genes and based on the presence of *qnr* genes.

### β-Lactamase contents of *E. coli* isolates

The strains were selected based on the most beta-lactams resistant (Table 2). They were resistant to antibiotics from at least three different class of beta-lactams or/and resistant to III’rd generation of cephalosporins. Based on the phenotypic analysis only two strains from whole collection (1.5%) were identified as ESBL producers and two other strains (1.5%) were producers of AmpC. All the strains from Table 2 were *bla*_TEM_ positive and it was also the most commonly identified gene present in 116 (91%) isolates (data not shown). It is worth adding that all *bla*_TEM_ negative strains were sensitive to most beta-lactam antibiotics used, primarily amoxicillin. The next most frequently occurring gene was *bla*_CMY-2_ (19.5% of studied strains), but only one strain of them was producer of AmpC beta-lactamase (Table 2). The rest of them (*bla*_CMY-2_ positive) were mostly resistant to at least one beta-lactam antibiotic, but they were not detected as ESBL strains. Only one strain (*E. coli* No 87) possessed *bla*_OXA_, but despite its resistance to most of the analyzed beta-lactam antibiotics (except amoxicillin/clavulanate and imipenem), it was not detected as ESBL-positive. Also, one other ESBL-negative strain possessed *bla*_SHV_ (*E. coli* No 108) and it was resistant to one beta-lactam antibiotic (ceftazidime). *bla*_CTX-M-1_ gene was not detected in any strains.

**Table 2:**
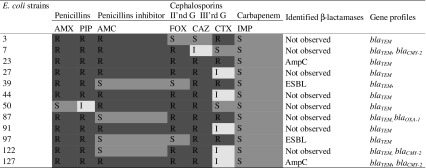
The correlation between phenotypic resistance to beta-lactams (the strains were selected based on the resistance to antibiotics from at least 3 different class of beta-lactams or/and resistant to III’rd generation of cephalosporins) and ESBL-connected genes identified in the studied collection of *E. coli* strains

*AMX* amoxicillin, *PIP* piperacillin, *AMC* amoxicillin/clavulanate, *FOX* cefoxitin, *CAZ* ceftazidime, *CTX* cefotaxime, *IMP* imipenem, *R* resistance, *S* sensitive, *I* intermediate

### Genes associated with aminoglycoside and sulfonamide resistance in *E. coli* strains

Further, the prevalence of genes connected with the resistance to aminoglycosides and sulfonamides was analyzed among the same clinical uropathogenic *E. coli* strains. The majority of studied bacteria (78% of strains) were *aac(3)-II* positive. These results did not correlate with resistance to aminoglycosides. Both group of strains resistant or sensitive have or have not carried *aac(3)-II* gene. We also analyzed the occurrences of *sul* genes. We wanted to see if there is any correlation between the phenotypic resistance to trimethoprim and/or cotrimoxazole and the occurrences of *sul* genes. The most popular gene in the studied collection of *E. coli* strains was *sul2—96*% of the studied strains were positive. Also, *sul1* was popular in 86% of these strains, but only 33% of the studied strains possessed *sul3*. Sensitive strains were positive for at least one *sul* gene (*sul1, sul2, sul3*). These results also did not correlate with resistance to sulfonamides.

### Genetic association with quinolone resistance in *E. coli* strains

**Fig. 1:**
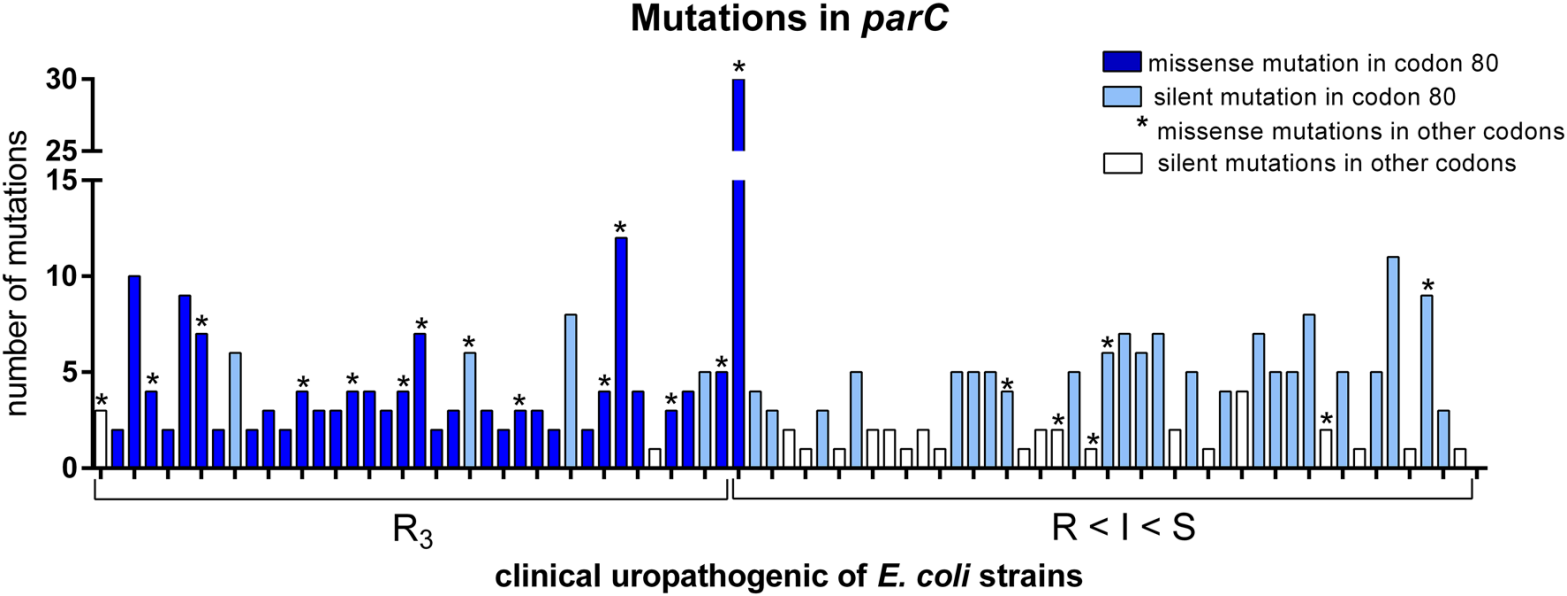
Characteristics of identified mutations in the *parC* gene of the studied uropathogenic *E. coli* strains The diagram was made using GraphPad Prism6

**Fig. 2:**
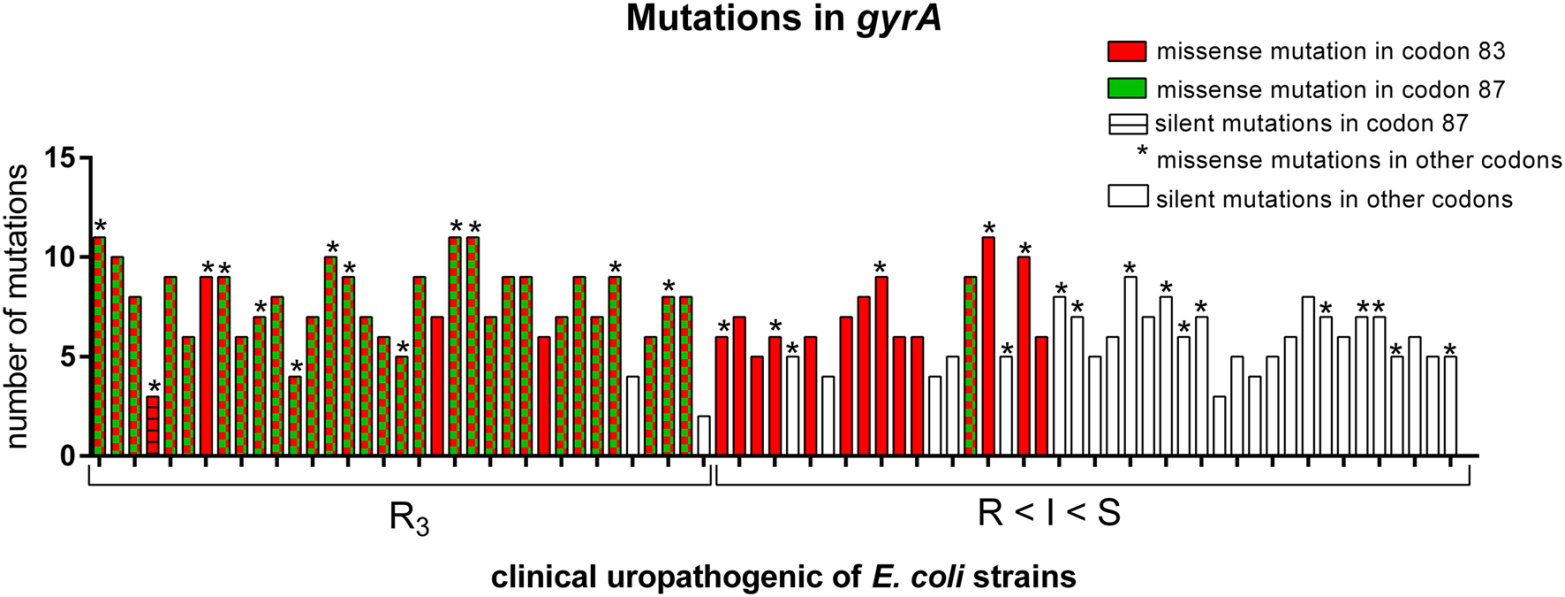
Characteristics of identified mutations in the *gyrA* gene of the studied uropathogenic *E. coli* strains The diagram was made using GraphPad Prism6

**Fig. 3:**
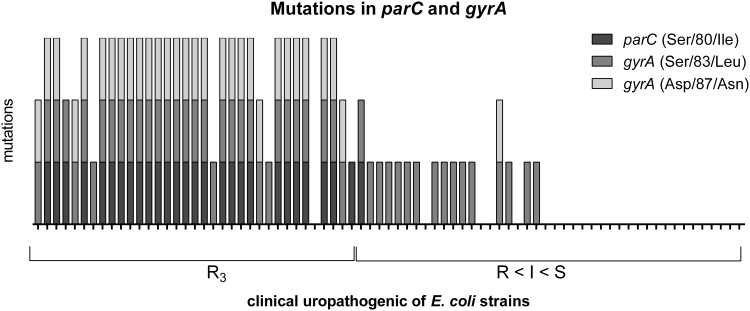
Comparison of occurrence of specific mutations in the *parC* and *gyrA* genes of the studied uropathogenic *E. coli* strains The diagram was made using GraphPad Prism6

The mutations in *parC* and *gyrA* were also investigated among the studied *E. coli* strains. The point mutations correlated with phenotypic resistance to fluoroquinolones are well known as Ser/80/Ile in *parC* and also Ser/83/Leu and Asp/87/Asn in *gyrA* [37, 41, 42]. They were likewise recognized in this study (Fig. 3). The strains were divided into two groups: R3—strains resistant to ciprofloxacin, norfloxacin, ofloxacin, and strains with decreasing resistance to fluoroquinolones (R < I < S). The strains from the second group were arranged on the basis of changing susceptibility: from resistance to 2 or 1 quinolones (R), intermediate sensitivity (I) to sensitivity to all three quinolones (S). The most common were silent point mutations in different codons—transversion and transition in *parC* and mainly transition in *gyrA*. All strains from R3 group were positive for specific missense mutations mentioned above. Ser/80/Ile mutation in *parC* and then Ser/83/Leu and Asp/87/Asn mutations in *gyrA* were the most frequently identified. The *parC* gene had more frequent silent mutations in comparison to *gyrA* (Fig. 1). Additionally, silent mutations in codon 80 of *parC* were typical in the second group of strains (R < I < S). In contrast, the silent mutation in the hot spot of *gyrA* was observed only in one *E. coli* strain No 9 (Asp/87/Asp). On the other hand, generally there were more mutations in the *gyrA* gene compared to the *parC* gene (Fig. 2). Further, in case of *gyrA* gene—the occurrence of the Asp/87/Asn missense mutation always correlated with the occurrence of the Ser/83/Leu mutation. Ser/83/Leu mutation occurred alone in the case of strains from R < I < S group. Strains which were the most sensitive to fluoroquinolones possessed only missense and/or silent mutations in other codons.

Additionally, the strains were analyzed based on the presence of *qnr* genes and despite many attempt of optimizing PCR procedures, the results remained negative.

## Discussion

The problem of bacterial antibiotic resistance belongs to the priorities of World Health Organization concerning threat to human health. The widespread use of antibiotics often without the antibiotic susceptibility testing is one of the reasons for the emergence of multidrug resistant pathogens, which seriously impedes therapeutic activities [43, 44]. This can also hinder other therapeutic successes as infectious complications appearing in patients undergoing chemotherapy for cancer or dialysis for renal failure. The effectiveness of secondary infections treatment is crucial also in surgery, especially organ transplantation [1, 45, 46]. Instead, urinary tract infections (UTIs) belong to the most common human infections in both hospital and community settings, where antibiotics are also usually applied [47]. Approximately every second woman and every twentieth man will suffer from UTI in their lifetime. Uropathogenic *Escherichia coli* (UPEC) is the primary agent causing uncomplicated and complicated UTIs [2, 47, 48]. Therapeutic difficulties result largely from the quick spread of multidrug resistance (MDR) among them. This problem concerns the large group of beta-lactam antibiotics but also other compounds, such as fluoroquinolones, aminoglycosides, etc., which are often used in UTI treatment [30]. The mechanisms of bacterial antibiotic resistance are often associated with the mobile properties of a genome, especially with horizontal gene transfer, the presence of plasmids or genomic mutations [6, 7, 49]. The genetic markers of bacterial antibiotic resistance are often described in the literature. The prevalence and differentiation of these genetic profiles vary depending on the countries, antibiotic policy, source and year of bacteria isolation.

In this study, we analyzed 14 genes related to resistance to antibiotics belonging to four different classes. We would like to check the relationships between genotypic and phenotypic resistance among uropathogenic *Escherichia coli* strains isolated from central Poland (Łódź). The obtained results represent the characteristic of the bacterial population from 10 years ago, which allowed us to look at the potential changes currently observed in a similar bacterial population. At present, the hot topic is ESBL strains. Among the studied bacterial collection we identified approx. 1.5% of ESBL-producing strains and 1.5% AmpC-producing strains, which was the standard level in those days (2005–2007). The situation in Poland seems to have been relatively stable for 10 years (at present approx. 2% ESBL of UPEC strains), but in many other countries the situation is much worse [3, 50–53]. However, the increase in bacterial antibiotic resistance is still observed. Considering the genetic background, these strains (ESBL- and AmpC-producing) carrying *bla*_TEM_ gene and additionally *bla*_CMY-2_ was present only in one AmpC-producing *E. coli* strain, although *bla*_CMY-2_ belongs to a small family of plasmid-mediated AmpC-like enzymes [54]. The remaining *bla*_CMY-2_ positive strains did not correlate with the production of AmpC beta-lactamase. The mechanisms of resistance against beta-lactam antibiotics seem to be the most complicated and differential. This is also clearly visible in the case of genetic background analysis. Considering that the vast majority of the isolates were ESBL-negative despite the presence *bla*_TEM_ in most strains (91%), it may be assumed that the *bla*_TEM_ genes encoded broad-spectrum enzymes, most likely TEM-1. TEM-1 is the major determinant of *E. coli* resistance to amino-penicillins and the most common plasmid-encoded β-lactamase; it is estimated that this enzyme occurs in approximately 50% all of *E. coli* clinical isolates [12, 13, 55]. As it was mentioned above, the *bla*_CMY-2_ was the next most common gene in the studied bacterial collection (19.5%). The *bla*_CMY-2_ has been also often identified in *E. coli, Klebsiella* sp. and *Salmonella* spp. from different sources in the United States, Greece and Algeria [54, 56–58]. The prevalence of this gene did not correlate with the resistance to β-lactams in our study. In the studied *E. coli* collection the other genes were sought, too. Only one strain possessed *bla*_OXA-1_ and despite its resistance to all analyzed beta-lactam antibiotics (except amoxicillin/clavulanate and imipenem), it was not detected as ESBL-positive. A similar prevalence of *bla*_OXA-1_ was observed at present by other authors [59, 60]. Also, one other strain possessed *bla*_SHV_ (*E. coli* No 108) and it was resistant to only one beta-lactam antibiotic (ceftazidime), which may suggest the presence of only broad-spectrum β-lactamase type SHV-1 or SHV-11 [61]. The SHV-2, SHV-5, SHV-7 or SHV-18 belong to the common ESBL variants that have been often observed in Poland [12, 13]. The *bla*_CTX-M-1_ gene was not detected in any strains, despite the first strain producing β-lactamase, CTX-M-3 was identified originally in Poland in 1996 [11], being the far predominant ESBL type in the country [11, 13]. Analyzing the occurrence of the bla_CTX-M-1_ gene, as reported in 2006, is not one of the most common genes in Poland like in the case of the genes encoding beta-lactamase from the CTX-M group: CTX-M-3, CTX-M-15, CTX-M-2 [62]. According to this, the lack of the *bla*_CTX-M-1_ gene in the collection of *E. coli* strains is not surprising. However, the distribution of the studied genes is very varied depending on the region, country, and the year of the strains isolation. The literature shows that CTX-M enzymes were identified in different locations in the second half of the twentieth century, among others, in Argentina, Israel, and Paraguay [62]. In Europe, *bla*_CTX-M-1_ genes were first identified in 1989 in Germany [62]. Results of antibiotic resistance from 2014 presented by Ojdana et al. [13] have shown that *bla*_CTX-M-15_ genes were identified in all of the 12 analyzed *E. coli* strains from a Polish patient (Białystok) and only two strains were positive for *bla*_TEM-1_ and *bla*_SHV_ genes. Bailey et al. [55] presented antibiotic resistance among the collection of commensal *E. coli* strains. 35% of these strains were ampicillin-resistant and containing the *bla*_TEM_ gene. In 2012, Korzeniewska et al. [63] published studies about the antibiotic resistance of *E. coli* strains from different sources (hospital and communal wastewater, river, air) and thereabouts 30% of studied strains were ESBL positive. In these strains genes *bla*_CTX-M-1_, *bla*_CTX-M-3_, *bla*_CTX-M-5_, *bla*_CTX-M-15_ were identified as the most common. Winokur et al. [14] analyzed the presence of the *bla*_CMY-2_ gene in *E. coli* strains isolated from people and animals in the USA. In the case of strains resistant to cephalosporins, *bla*_CMY-2_ was identified in 33% of human isolates and 94.8% of animal isolates [14]. It must be noted that the occurrence of antibiotic resistance genes usually differ between people and animals and they are determined by many other factors. It is very well known that bacteria can induce a lot of mechanisms against drugs, so the specific genes often do not correlate with the phenotypic antibiotic resistance, for example, structural changes at the site of the drug’s action or change of the action point for the antibiotic [43, 44]. Additionally, the efflux pumps system AcrAB-TolC, AcrEF-TolC, AcrABC-TolC is also often described in case of *E. coli* phenotypic resistance to beta-lactam antibiotic [64]. As we can see, the distribution of the analyzed genes is not correlated with the year in a global coverage, but in the Polish view we can see the rise of the number of antibiotic resistance genes in *E. coli* strains [10].

Taking into consideration the resistance to other antibiotics, aminoglycosides and sulfonamides also play a significant role during UTI treatment. The *aac(3)-II* is described as the most correlated gene with aminoglycosides resistance, which was not confirmed in our results. Both group of strains, resistant or sensitive, have or have not carried the *aac(3)-II* gene. Therefore, it should be stated that *aac(3)-II* cannot play the role of a marker for resistance to aminoglycosides or at least for one of them. A similar high prevalence of that gene was observed in other studied collection of *E. coli* isolated from Europe [36, 65, 66]. We also analyzed the occurrences of *sul* genes which are responsible for resistance to sulfonamides by changed activity of DHPS (dihydropteroate synthase). This enzyme shows affinity to PABA and when it is encoded by *sul* it remains insensible to sulfonamides. Trimethoprim, which we used in our study, binds to dihydrofolate reductase and inhibits the reduction of dihydrofolic acid (DHF) further upstream in the same pathway [67]. We wanted to check if there is any correlation between the phenotypic resistance to trimethoprim and the occurrences of *sul* genes—there was none. Furthermore, the presence of the studied *sul* genes also correlated with resistance to cotrimoxazole. These genes were very common for the studied *E. coli* strains, even in the case of strains sensitive to trimethoprim or cotrimoxazole, which can only mean that sulfonamides may enhance the expression of resistance to these antibiotics. The results presented by other authors show a similar distribution of these genes [68, 69]. Completely different results were presented by Mazurek et al. [70] where the presence of the studied genes was significantly lower. However, those *E. coli* strains were isolated from animals in Poland which could probably be an important reason for the differences [70]. The rare occurrence of *sul3* may prove that the synthesis of modified DHPS due to the presence of *sul3* gene has recently appeared in resistant bacteria strains [71]. This confirms the high incidence in this group of questionable and negative results obtained by PCR. Sulfonamides therapy is used in UTI as a combination of sulfamethoxazole with trimethoprim. This was the dominant therapy for UTI between 1995 and 1996. At present, due to the increasing prevalence of resistance to trimethoprim-sulfamethoxazole among *E. coli* strains, it should not be the first method of choice in the treatment of *E. coli* infection [47].

The most obvious results were obtained by analyzing the point mutations in *parC* and *gyrA* genes. Ser/80/Ile in *parC* and also Ser/83/Leu and Asp/87/Asn in *gyrA* give a strong correlation with phenotypic resistance to quinolones [37, 41, 42]. Our results confirm these findings (Fig. 1); however, there can be some other possible mechanisms involved in quinolones resistance, like the permeability effect, efflux pumps, and the decreased availability of quinolones at the target site can also be involved [9, 72, 73]. Looking more closely at the results we observed a specific tendency. The group of strains with reduced susceptibility to quinolones (R < I < S) carried a lot of atypical mutations (Figs. 2, 3). The silent mutations in the hot spot were characteristic for *parC*, and missense mutations in other codons were characteristic for *gyrA*. It can play a predictive role of imminent phenotypic resistance to fluoroquinolones and makes it suitable for epidemiological studies [74]. Silent mutation have also been identified by other authors [73, 75–77]. It could be interesting to observe the genetic background during the process of acquiring resistance to fluoroquinolones. This can be related to the accumulation of mutations because of the low specificity of fluoroquinolones action. Also, the antibiotics, especially fluoroquinolones can induce the response of SOS systems, which can be responsible for DNA changes in bacteria genome [78, 79]. There is some evidence that silence mutations might cause a phenotypic effect, they can especially have an influence on the regulation of transcription [80–82]; possibly they can also change the affinity of the hot spot to fluoroquinolones. Nevertheless, we can conclude that the hot spot of *parC* is more specific but less sensitive to fluoroquinolones (more silent mutations), whereas *gyrA* conversely—a lot of missense mutations give the phenotypic effect but not in the hot spot of *gyrA*. So, in this case, two hot spots were evolved. We have not identified more other specific mutations, but in the literature other mutations correlated with phenotypic resistance to quinolones were identified. These mutations were described generally in *gyrA* and *parC* genes, for example as new and rare mutations in *gyrA*—Ser/83/stop, Asp/82/Asn, Gly/81/Asp, Asp/82/Gly, Ser/431/Pro in resistant *E. coli* strains [83].

Additionally, none of the strains possessed the *qnr* genes. This similar low prevalence is still relevant in most cases [84–87]. Mammeri et al. in 2005 [72] published an analysis of the *qnr* gene in a collection of 297 of nalidixic-acid resistant *E. coli* strains. In this collection, only 1 strain with the *qnr* gene was identified. The low level of identification of the *qnr* gene can be due to the weak expression of the Qnr determinant [72]. Some authors present dissimilar results [88–90]. It probably depends on the local distribution of Qnr plasmid.

To conclude, the genetic background is not sufficient for identifying bacterial antibiotic resistance. This kind of analysis can play a role for predicting resistance and it may mark the high or low risk of the emergence of resistance. However, quinolones resistance is very strongly dependent on the specific mutations. As we can see, the distribution of the analyzed genes is very differentiated and shows a high adaptive potential of bacteria to a toxic (antibiotic) environment.
